# Exome sequence analysis in consanguineous Pakistani families inheriting Bardet‐Biedle syndrome determined founder effect of mutation c.299delC (p.Ser100Leufs*24) in *BBS9* gene

**DOI:** 10.1002/mgg3.834

**Published:** 2019-07-11

**Authors:** Muhammad Muzammal, Muhammad Zubair, Sophie Bierbaumer, Jasmin Blatterer, Ricarda Graf, Aisha Gul, Safdar Abbas, Muhammad Badar, Ansar Ahmad Abbasi, Muzammil Ahmad Khan, Christian Windpassinger

**Affiliations:** ^1^ Gomal Centre of Biochemistry and Biotechnology Gomal University Dera Ismail Khan Pakistan; ^2^ Department of Zoology Mirpur University of Science and Technology (MUST) Mirpur Pakistan; ^3^ Institute of Human Genetics Medical University of Graz Graz Austria

**Keywords:** BBS9, exome sequencing, founder effect, frameshift mutation, Pakistani family

## Abstract

**Background:**

Bardet‐Biedl syndrome (BBS) is characterized by a heterogeneous phenotypic spectrum of retinopathy, intellectual disability (ID), obesity, polydactyly, and kidney dysfunctions as the major clinical features. Genetic investigations have reported 21 BBS genes, the products of which are mostly located at the centrosome, basal body or the ciliary transition zone.

**Methods:**

In the present genetic report, we analyzed two apparently unrelated consanguineous BBS families from Dera Ismail Khan (D.I.Khan) district, Pakistan. Genetic mapping was performed using Whole exome sequencing and Sanger sequencing.

**Results:**

Whole exome sequencing identified a recently reported single base deletion NM_001033604.1:c.299delC in the fourth exon of *BBS9* in both families. The identified frameshift mutation is predicted to cause premature truncation of the expressed protein (p.Ser100Leufs*24). This mutation has previously been mapped in a consanguineous Pakistani family; therefore this is the second report of this particular mutation in two additional BBS families originating from different locations.

**Conclusion:**

We speculate the evolutionary significance of this mutation and assume its strong founder effect in the Khaisoori tribe of D.I.Khan. Based on these findings, we suggest developing a molecular diagnostic test that may be used for premarital and prenatal screening of families at risk of BBS.

## 1 INTRODUCTION

Bardet‐Biedl syndrome (BBS; MIM# 209,900) is a multisystematic autosomal recessive disorder. The primary clinical manifestations of BBS include retinal degeneration, hypogonadism, digit abnormalities, truncal obesity, intellectual impairments, and renal anomalies. Secondary features may include speech impediments, behavioral abnormalities, orodental anomalies, cardiovascular malformations, syndactyly/brachydactyly, mild hypertonia, ataxia, diabetes mellitus type 2, hepatic malfunction, craniofacial dysmorphism, and eye abnormalities like, cataracts and strabismus. At least 21 genes are associated with BBS (OMIM database, accessed on 26‐09‐2018), among which *BBS1* (MIM# 209901), *BBS10* (MIM# 610148), *BBS2* (MIM# 606151), *BBS9* (MIM# 607968), *MKKS* (MIM# 604896) and *BBS12* (MIM# 610683) are the most common. Although demographic data on BBS prevalence in Pakistan is not yet available, genetic studies have so far reported eight BBS genes including *ARL6* (MIM# 608845)*, BBS1*, *BBS2, BBS5* (MIM# 608845), *BBS9* (MIM# 607968), *BBS10*, *BBS12,* and *TTC8* (MIM# 608132) in Pakistani families (Maria et al., 2016).

The prevalence rate of BBS varies among different ethnicities worldwide. In North America and Europe, BBS affetcs 1 per 140,000–160,000 live births. However, its incidence is extremely high with ratios of 1:18,000, 1:13,500, and 1:3,700 in geographically isolated communities of Newfoundland, Kuwaiti Bedouins and The Faroe Islands, respectively. Nonetheless, the additional contributing factor of elevated BBS incidence in the Kuwaiti population is probably the high ratio of consanguinity (Maria et al., 2016).

This study was aimed at exploring the genetics of BBS phenotypes in two apparently nonrelated consanguineous families of Pashtoon origin, recruited from a small village in Khyber‐Pukhtunkhwa (KPK) province of Pakistan. Exome sequence analysis in both families revealed a previously reported single base deletion mutation in *BBS9* [c.299delC (p.Ser100Leufs*24)], which suggests its founder effect in the Khaisoori tribe of D.I.Khan city in KPK.

## 2 MATERIALS AND METHODS

### 2.1 Family recruitment

Two apparently unrelated consanguineous families of Pashtoon origin exhibiting the BBS phenotype were enrolled in the present genetic study. Pedigree information of both families was procured to explain the consanguineous relationship and degree of consanguinity. Both families were recruited from the Rehmani‐Khail village, a remote area in D.I.Khan city in the KPK Province of Pakistan. Prior approval for the study was obtained from the ethical review board of Gomal University, D.I.Khan, Pakistan. Participants or their guardians were briefed on the study scheme, and written consent was obtained.

### 2.2 Clinical assessment

Details of relevant BBS associated clinical phenotypes were documented using a self‐designed questionnaire by comprehensively evaluating the apparent features in all patients. Subsequently, peripheral blood samples from cooperative affected and unaffected participants of the study were collected and DNA was extracted using standard laboratory protocols. Additionally, the peripheral blood samples were subjected to certain biochemical tests, such as lipid profile, liver function tests and renal function test. These spectrophotometric assays were conducted using a chemistry analyzer (Macrolab, Germany). Furthermore, profile photographs of certain patients were obtained to document and publish the patients' phenotype.

### 2.3 Exome sequencing and mutation analysis

To map the causative gene manifesting BBS in both families, one patient from each family was selected for whole exome sequencing. Sample library preparation was performed with Nextera Rapid Capture Exome Kit (Illumina, USA) and sequenced using the NextSeq550 (Illumina, USA) at the Institute of Human Genetics, Medical University of Graz, Austria. Raw sequence data were aligned to the reference genome using the BaseSpace applications to generate BAM and VCF files. Thereafter, variant filtering was performed using VariantStudio Software (Illumina, USA). Primarily, data were filtered for homozygous, nonsynonymous SNPs and InDel variants with an allele frequency below 1% in the coding regions of known BBS genes.

In addition to this, gene ontology and protein function‐based bioinformatics analysis was performed using PhenIX software (Zemojtel et al., 2014)]. Subsequently, segregation analysis of potentially pathogenic variants in the whole family was done using Sanger DNA sequencing.

### 2.4 Results

**Figure 1:**
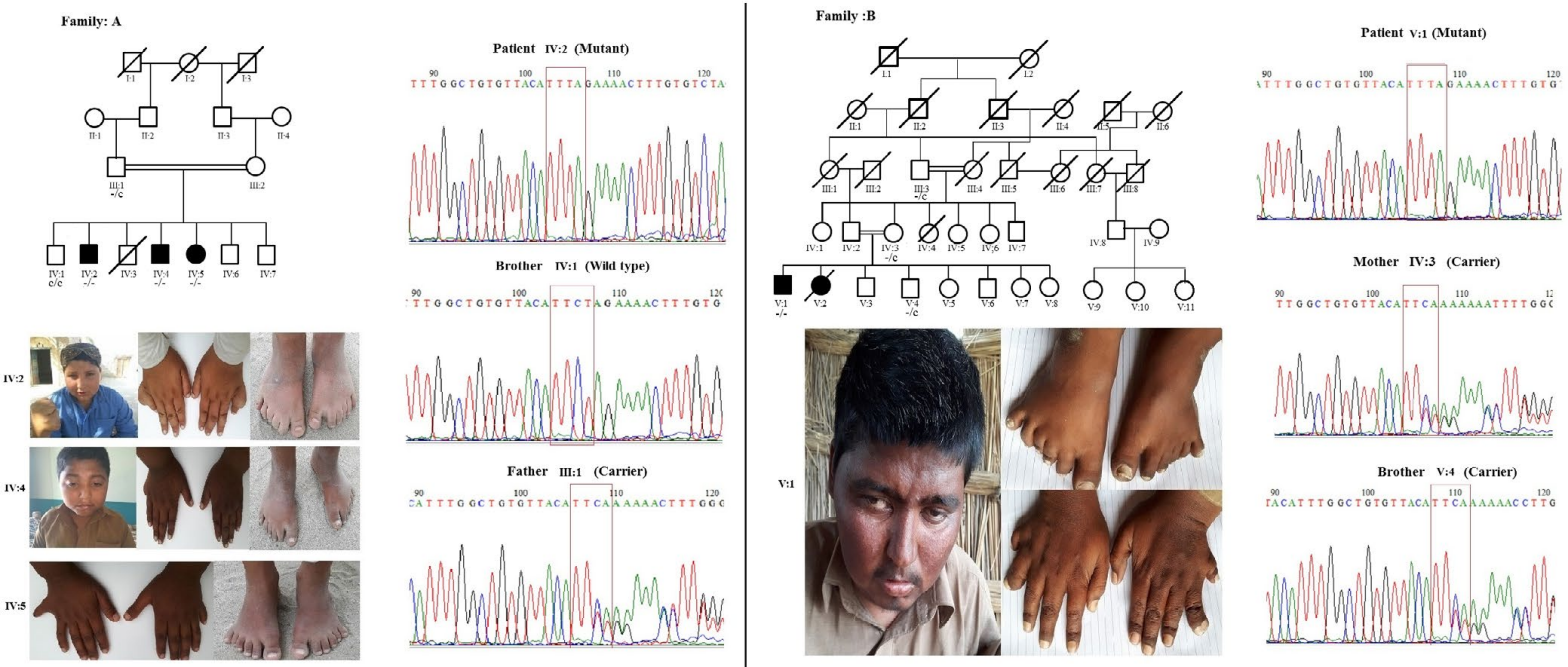
Depicted are the pedigree, patient's photographs and representative Sanger sequencing chromatogram traces at the position of the variant for family A and B, respectively. Pedigrees show the genotype status of analyzed individuals, represented as ‐/‐ (mutant), ‐/c (carrier) and c/c (WT). The position of the deletion is framed in red in the chromatogram

This study focuses on two consanguineous families of Pashtoon origin from the Rehmani‐Khail village in D.I.Khan city of Pakistan, exhibiting autosomal recessive BBS. Although, both families ethnically belong to the Rehmani‐Khail tribe, they were unable to establish a close relationship with each other or previously published family from Khan, Mohan, et al. (2016) and Khan, Muhammad, et al. (2016). All the BBS children were born from asymptomatic parents with first cousin relationship (Figure 1).

### 2.5 Clinical outcomes

Patients from both families exhibited postaxial polydactyly of hands and feet, obesity and intellectual disability (ID) as a common phenotype. Gross clinical analysis confirmed diagnosis of BBS syndrome in all cases. All patients from family A showed a mild level of ID, while patient/V from family B had severe ID. Severely reduced visual acuity was only observed in one affected person from family B, along with strabismus of right eye, however this phenotype was not observed in patients from family A. Speech was quite developed in all patients of family A, but patient/V from family B was unable to speak. Analysis of facial photographs did not reveal facial dysmorphology (Figure 1). Patients from both families revealed hexadactyly (central polydactyly) of both feet, however, their hands exhibited syn‐hexa‐dactyly (see table and figure for details). Furthermore, there were no visceral organ defects observed in either family. The summarized clinical features, along with additional information, are presented in Table 1. We interviewed the family elders from both familes of the current study (Rehmani‐khail), and the family described in the previously published study from Khan, Mohan, et al. (2016) and Khan, Muhammad, et al. (2016) (Khanokhail tribe). On the basis of their statements a distant kinship with common ancestors, i.e. the Khaisoori tribe, seems possible.

**Table 1:** Clinical summary of affected individuals of family A and B described in the current study, as well as the previously published family from Kahn, Mohan, et al. (2016) and Kahn, Muhammad, et al. (2016)

Family IDs	A			B	Published family from Khan, Mohan, et al. (2016) and Khan, Muhammad, et al. (2016)
Pedigree ID	IV−4	IV−2	IV−5	V−1	
**Clinical features**					
Gross diagnosis	BBS	BBS	BBS	BBS	BBS
Age	~10–11	~13	~8	~24	—
Gender	male	male	female	male	—
Occipitofrontal circumference	51 cm	53 cm	51 cm	52 cm	Normal head circumference
Microcephalic feature	No	No	No	No	No
Height	4 feet	5–1"	3–10"	5 feet	—
BMI*	30.5 (obese)	27 (obese)	26.9 (obese)	27.9 (over weight)	Obese
Age of disease onset	Congenital	Congenital	Congenital	Congenital	Congenital
Involvement of environmental factors	No	No	No	No	No
Behavioral expression	Hyperactive	Hyperactive	Hyperactive	Lethargic	Hyperactive
Communication ability	Normal	Weak	Normal	Nil	Weak
Growth condition	Normal	Normal	Normal	Weak	—
Epileptic shock	No	No	No	No	—
Gait	Wide	Wide	Wide	Wide	—
Attention	Yes	Yes	Yes	No	Yes
Muscle degeneration	No	No	No	No	No
Abnormal spine curvature	No	No	No	No	No
Liver status (LFTs)	Abnormal (high ALT/SGPT & Alk. Phosphate)	Abnormal (high ALT/SGPT & Alk. Phosphate)	Abnormal (high ALT/SGPT & Alk. Phosphate)	Abnormal (high ALT/SGPT Alk. Phosphatase)	Apparently normal
Kidney status (RFTs)	Normal	Normal	Normal	Boarder‐line	Abnormal
Lipid profile	High (Abnormal triglyceride)	High (Abnormal triglyceride)	Normal	Normal	—
Facial morphology	Normal	Normal	Normal	Normal	Normal
Webbing of neck	No	No	No	No	No
Polydactyly	Hexadactyly of both feet and hands	Hexadactyly of feet, syn‐hexa‐dactyly of both hands	Hexadactyly of both feet and hands	Hexadactyly of feet, Hexa‐dactyly of right hand, Syn‐hepta‐dactyly of left hand	Hexadactyly of feet, syn‐polydactyly of both hands
Syndactyly	No	Yes	No	Yes	Yes
Dental anomalies	No	No	No	No	No
Retinitis Pigmentosa	Yes	Yes	No	Yes	Yes
Eye sight	Weak	Weak	Normal	Weak	Weak
Nystagmus	No	No	No	Yes	—
Color blindness	No	No	No	No	—
Strabismus	No	No	No	No	Observed in male patient
Deafness	No	No	No	No	Impaired in one patient
Diabetes	No	No	No	No	No
Digestive system functionality	Normal	Normal	Normal	Normal	Abnormal
Cardiac status	Normal	Normal	Normal	Normal	Normal
Renal status	Normal	Normal	Normal	Normal	Abnormal

Note:

“BMI calculator” available on CDC (website www.cdc.gov) is used for BMI calculation.

### 2.6 Genetic outcomes

Whole exome sequencing identified a recently reported single base deletion c.299delC in the fourth exon of the *BBS9* gene in both family A and B. This frameshift mutation is predicted to cause premature truncation of the protein product (p.Ser100Leufs*24), possibly leading to a loss of part of its N‐terminal and all of its C terminal domains. In both familes the detected mutation co‐segregated with the disease phenotype.

Additionally, exome‐wide SNP analysis for HBD mapping revealed both families sharing common haplotype between markers rs201037213 and rs2240350 around the *BBS9* locus on chrosomsome 7.

## 3 DISCUSSION

Bardet‐Biedl syndrome (BBS) is an autosomal recessive disorder that affects many tissues of the body simultaneously. According to BBS diagnosis criteria, the patients should either have four major features or three major and two minor features for positive diagnosis of BBS. Genetic investigations have reported 21 BBS genes so far, among them *BBS1* and *BBS10* are highly reported in Europe and North America, while *BBS2, BBS4, BBS5,* and *BBS12* are prevalent in the Middle East and North Africa (Nikkhah et al., 2018). Nonetheless, to date, 18 Pakistani families harboring eight genes have been reported to be involved in BBS (Maria et al., 2016).

In this study, whole exome sequencing in two families revealed a previously reported frameshift mutation p.Ser100Leufs*24 in *BBS9*. The identified mutation is predicted to severely truncate the BBS9 protein and completely remove its C‐terminus domain. Predictably, the defective mRNA may be removed through mRNA degradation as in an analogous case of protein truncating nonsense mutation p.Q597* reported by Maria et al., (2016).

Bardet‐Biedl syndrome‐9 (*BBS9*) gene is situated on chromosome 7p14.3, and consists of 22 coding exons (NM_198428.2), which encodes an 887 amino acid long protein. This protein contains two functional domains that is, PTHB1, N‐terminus and C‐terminus domains (UniProt and InterPro databases). Physiologically, BBS9 is involved in various biological processes, that is, cilium assembly (biogenesis/degradation), fat cell differentiation, protein localization to the cilium, protein transport response to stimulus and visual perception. Gene expression studies have determined its expression in adult cardiac tissue, skeletal muscle, lung, liver, kidney, placenta, brain (Adams, Rosenblatt, & Suva, 1999). Therefore, defective BBS9 protein exhibits multi‐tissue disorder. Morphologically, BBS9 is the structural component of BBSome complexes, which are composed of eight highly conserved BBS proteins that is BBS1, BBS2, BBS4, BBS5, BBS7, BBS8, and BBS9 and BBIP10 (Scheidecker et al., 2014). This complex is involved in the biogenesis of primary cilia, microtubule‐based projections found on nearly all types of vertebrate cells. The role of primary cilium has been explored in signal transduction, especially G protein‐coupled receptors (Berbari, Lewis, Bishop, Askwith, & Mykytyn, 2008). The physiology of BBSome has been studied in Bbs2 and Bbs4 knockout mice, where the somatostatin receptor type 3, a G protein‐coupled receptor located in the ciliary membrane, fails to localize in the primary cilium (Berbari et al., 2008).

Although, genetic studies have reported 52 mutations (HGMD, accessed on 9.11.2018) in the *BBS9* genes, however, only two mutations been reported in Pakistani families so far. The first *BBS9* report describes a c.299delC mutation (Khan, Mohan, et al., 2016; Khan, Muhammad, et al., 2016), while the second mutation c.1789C > T (p.Q597*) was reported by Maria et al., (2016). Both reported mutations are predicted to cause protein truncations. In a c.299delC linked comparative phenotyping analysis of the three recruited families (two from this study and one from Khan, Mohan, et al., 2016; Khan, Muhammad, et al., 2016), it was observed that patients exhibit syn‐polydactyly of hands, obesity, intellectual disability and ophthalmic consequences as common clinical features. Hence, it is assumed that these phenotypic features could be the hallmarks of mutation c.299delC in *BBS9*. Moreover, the same deletion mutation (c.299delC) had previously been mapped in a consanguineous Pakistani family ethnically belonging to Khano‐khail tribe from a nearby village in D.I.Khan city in Pakistan. Upon revisiting the previous & current families for exploring their ancestral history, it was discovered that both the Rehmani‐khail and Khano‐khail tribes belong to the Khaisoori tribe, according to an undocumented historic proof. Nevertheless, the subsequent mutation analysis confirms the body of evidence that both tribes are distantly related.

## 4 CONCLUSION

This study involved genetic dissection of two apparently unrelated BBS families from Rehmani‐Khail village of Pakistan. Exome sequence analysis determined a previously reported frameshift mutation c.299delC (p.Ser100Leufs*24). The same mutation was mapped by Khan, Mohan, et al. (2016) and Khan, Muhammad, et al. (2016) in a Pakistani family from a nearby village in D.I.Khan city. Hence, this study reports a founder mutation in the Khaisoori tribes of D.I.Khan. Genetic counseling was provided to the parents of both families in order to prevent the risk of BBS in further pregnancies.

## CONFLICT OF INTEREST

The authors declare that they have no financial as well as competing interests.

## AUTHORS' CONTRIBUTION

MM, MZ, SB, RG and JB collected the samples, performed experimental work, data analysis and drafted the manuscript. AG, SA, MB and AAA were involved in data analysis and manuscript drafting. MAK and CW designed the project, arranged sequencing data, performed data analysis and drafted the final version of the manuscript. All authors have read and approved the final version.
